# Development of an Accelerated Solvent Extraction-Ultra-Performance Liquid Chromatography-Fluorescence Detection Method for Quantitative Analysis of Thiamphenicol, Florfenicol and Florfenicol Amine in Poultry Eggs

**DOI:** 10.3390/molecules24091830

**Published:** 2019-05-13

**Authors:** Bo Wang, Xing Xie, Xia Zhao, Kaizhou Xie, Zhixiang Diao, Genxi Zhang, Tao Zhang, Guojun Dai

**Affiliations:** 1 College of Veterinary Medicine, Yangzhou University, Yangzhou 225009, China; yzwbo168@163.com; 2 Joint International Research Laboratory of Agriculture & Agri-Product Safety, Yangzhou University, Yangzhou 225009, China; 18252711481@139.com (X.Z.); 18352764521@163.com (Z.D.); zgx1588@126.com (G.Z.); zhangt@yzu.edu.cn (T.Z.); gjdai@163.com (G.D.); 3 Key Laboratory of Veterinary Biological Engineering and Technology, Ministry of Agriculture, Institute of Veterinary Medicine, Jiangsu Academy of Agricultural Sciences, Nanjing 210014, China; yzxx1989@163.com; 4 College of Animal Science and Technology, Yangzhou University, Yangzhou 225009, China

**Keywords:** poultry eggs, thiamphenicol, florfenicol, florfenicol amine, ASE, UPLC-FLD

## Abstract

A simple, rapid and novel method for the detection of residues of thiamphenicol (TAP), florfenicol (FF) and its metabolite, florfenicol amine (FFA), in poultry eggs by ultra-performance liquid chromatography-fluorescence detection (UPLC-FLD) was developed. The samples were extracted with acetonitrile-ammonia (98:2, *v/v*) using accelerated solvent extraction (ASE) and purified by manual degreasing with acetonitrile-saturated n-hexane. The target compounds were separated on an ACQUITY UPLC^®^ BEH C_18_ (2.1 mm × 100 mm, 1.7 μm) chromatographic column using a mobile phase composed of 0.005 mol/L NaH_2_PO_4_, 0.003 mol/L sodium lauryl sulfate and 0.05% trimethylamine, adjusted to pH 5.3 ± 0.1 by phosphoric acid and acetonitrile (64:36, *v/v*). The limits of detection (LODs) and limits of quantification (LOQs) of the three target compounds in poultry eggs were 1.8–4.9 µg/kg and 4.3–11.7 µg/kg, respectively. The recoveries of the three target compounds in poultry eggs were above 80.1% when the spiked concentrations of three phenicols were the LOQ, 0.5 maximum residue limit (MRL), 1.0 MRL and 2.0 MRL. The intraday relative standard deviations (RSDs) were less than 5.5%, and the interday RSDs were less than 6.6%. Finally, this new detection method was successfully applied to the quantitative analysis of TAP, FF and FFA in 150 commercial poultry eggs.

## 1. Introduction

Poultry eggs contain high levels of essential amino acids and vitamins as well as various major and trace elements required by the human body, and as a result they have become an increasingly popular consumer product [1]. Consumer demand for poultry products has promoted the growth of the poultry industry, and intensive farming has also increased the morbidity and mortality of poultry. To control the occurrence of diseases and reduce mortality in poultry, antibiotics are widely used to prevent poultry diseases, increase feed conversion rates, and promote animal growth [2].

Thiamphenicol (TAP) and florfenicol (FF) are synthetic chloramphenicols (CAPs) used as broad-spectrum antibiotics, and they have chemical structures and efficacies similar to that of CAP as well as good therapeutic effects on various bacterial strains common in poultry. These compounds are widely used in actual production [3]. The only difference between TAP and CAP is the structure of the substituents on their benzene rings. CAP has a nitro group on the phenyl ring, and TAP has a methyl sulfone group. However, TAP is much less toxic than CAP, its blood toxicity effects are reversible, and it does not cause aplastic anemia. On the other hand, TAP can inhibit the formation of red blood cells, white blood cells and platelets; it has a strong immunosuppressive effect; and it has weaker antibacterial effects than CAP, which limit its practical use [4,5,6]. Many countries use these compounds as veterinary drugs, but they are banned from use in food animals. FF is a fluorinated analogue of TAP with a molecular structure similar to that of CAP, but it lacks the nitro group on the aromatic ring. Studies have shown that this substituent is the key molecular characteristic of CAP causing dose-independent irreversible aplastic anemia in the human body [7]. However, FF can theoretically cause serious adverse reactions similar to those caused by CAP. Therefore, FF can only be used for the treatment of animal diseases [8]. FF shows the most potent antibacterial activity among CAP drugs, and it has many advantages (wide spectrum of antibacterial activity, good oral absorption, wide distribution in the body, high bioavailability, good safety profile, etc.), making it a broad-spectrum antibiotic with great potential for practical applications [9]. At present, the drug is on the market in many countries, and it is widely used in animal husbandry and aquaculture for disease prevention. However, reproductive toxicity tests have shown that FF has certain embryotoxic effects, and as a result the detection of its residue in animal foods such as livestock, poultry, and aquatic products has attracted increasing attention [10].

Because both TAP and FF have toxic side effects, the EU [11], US [12] and China’s Ministry of Agriculture [13] have established maximum residue limits (MRLs) for these compounds in poultry tissues (TAP: 50 μg/kg, FF: 100 μg/kg in muscle), and stipulated that the limit of FF residue in poultry tissues is based on the total amount of both the prototype drug (FF) and its metabolite, florfenicol amine (FFA), and that these drugs should not be detected in poultry eggs. Therefore, developing different detection methods to determine whether veterinary drug residues meet the legal requirements before the animal foods are marketed is of great importance. Establishing and improving the detection methods for TAP, FF and FFA residues in poultry eggs is also necessary.

At present, there are many reported methods for detecting TAP, FF and FFA residues, including methods based on high-performance liquid chromatography (HPLC) [14,15,16], gas chromatography (GC) [17,18], liquid chromatography-tandem mass spectrometry (LC-MS/MS) [19,20,21,22], and gas chromatography-tandem mass spectrometry (GC-MS) [23,24]. When using GC to analyze CAP drug residues, the target needs to be derivatized, making the analysis process cumbersome. The most widely used analytical method involves LC-MS, and although this method offers qualitative and quantitative (mass spectrometry) accuracy and high sensitivity, the instrument is expensive, and the detection cost is high. Meanwhile, fluorescence detection is commonly used for veterinary drug residues and environmental analysis because of its advantages of speed, ease of operation, and low cost of detection [14,25]. Therefore, developing a simple, fast and low-cost analytical method that meets the detection requirements is of great importance. Moreover, in the reported detection methods, the sample matrices used for analysis were generally animal tissues [15,26] or aquatic products [21,27], and there are few reports on detection methods for poultry eggs [14]. Xie et al. [14] established an HPLC-FLD method for the determination of TAP, FF and FFA residues in eggs and sample pretreatment using a liquid-liquid extraction method, ethyl acetate:acetonitrile:ammonium hydroxide (49:49:2, *v/v/v*) as an extractant, delipided in n-hexane. Based on previous research, a comprehensive method using ultra-performance liquid chromatography-fluorescence detection (UPLC-FLD) for the determination of TAP, FF and FFA residues in poultry eggs (hen eggs, duck eggs, goose eggs, pigeon eggs and quail eggs) is reported here. Compared with the previously studied HPLC-FLD method [14], the UPLC-FLD method has the advantages of fast analysis speed (detection time < 5 min), strong separation ability (recoveries were 80.1%–98.6%), high sensitivity, and low consumption of reagents. This study intends to use accelerated solvent extraction (ASE) as the sample pretreatment procedure to extract the target analytes from the samples, aiming to establish an ASE-UPLC-FLD method for the determination of TAP, FF and FFA residues in poultry eggs. This technique will provide a new, simple, inexpensive, highly efficient and rapid method for the detection of these analytes. In addition, the effects of ultrasonic extraction, vortex oscillation extraction, vortex oscillation + ultrasonic extraction and ASE extraction are compared in this study. Compared to other extraction methods, ASE was investigated as a novel alternative technology, which has the advantages of automation (saving time and human effort), consuming less reagents, higher recovery rate, and suitability for batch processing of samples.

## 2. Results and Discussion

### 2.1. Selection of the Chromatographic Column and Mobile Phase

**Figure 1:**
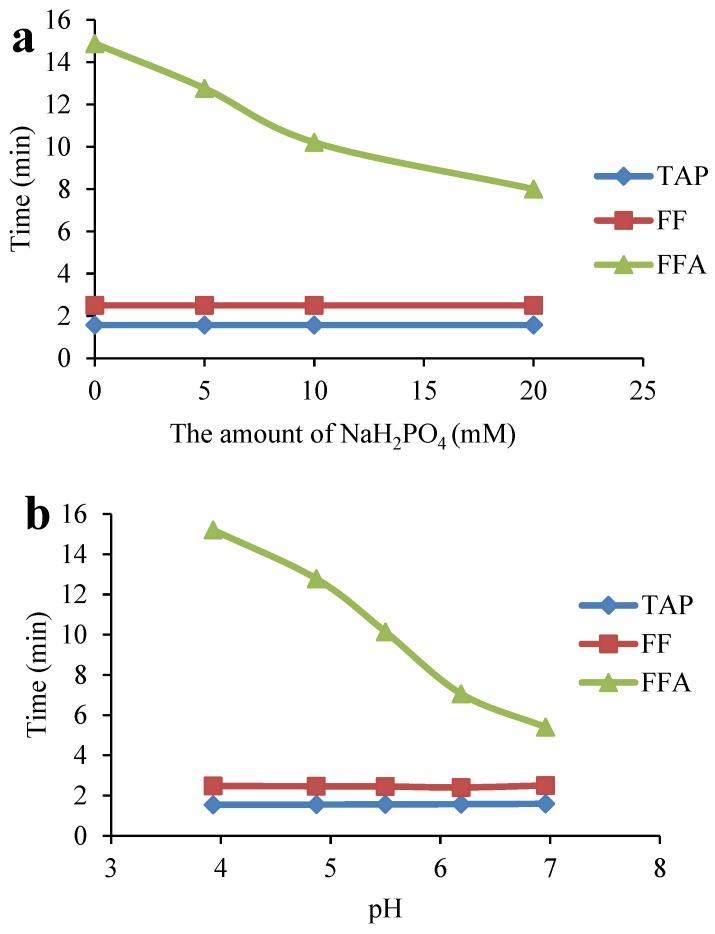
Effects of the amount of NaH_2_PO_4_ (**a**) and the mobile phase pH (**b**) on the retention times of the targets.

**Figure 2:**
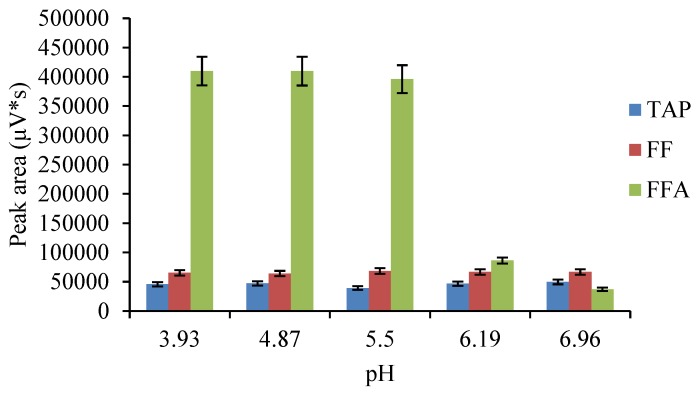
Effects of pH on the target responses.

The composition of the mobile phase and the type of chromatographic column have a substantial influence on the separation and peak shape of the analytes. Among the reported methods, the most commonly used columns for the detection of CAP drugs are C_18_ columns [28,29], produced by various manufacturers. Meanwhile, the ACQUITY UPLC^®^ BEH C_18_ (2.1 mm × 100 mm, 1.7 μm) column offers outstanding chemical stability, a wide range of pH conditions (pH 1-12) and a wide range of mobile phases, which provides a versatile and reliable separation technique for method development. Therefore, this study used an ACQUITY UPLC^®^ BEH C_18_ (2.1 mm × 100 mm, 1.7 μm) column as the analytical column. For the mobile phase, acetonitrile-water [16,30] mixtures are often used to determine CAP residues in LC-MS methods. FFA is a weakly basic substance that does not generally remain on the C_18_ column, and it elutes with the dead volume. A common solution to this is to add ammonium formate or ammonium acetate [27,31] to the mobile phase system to enhance the retention of FFA on the C_18_ column. When detecting CAPs with a fluorescence detector, to enhance the retention of FFA on a C_18_ column, an ion-pair reagent is usually added to the aqueous phase to react with the FFA to form a weakly bound ion pair, and the pH is adjusted with a buffer to keep the whole system weakly acidic and prevent dissociation of the ion pair [14,32]. Yang et al. [33] reported a liquid chromatography-fluorescence detection method for the determination of TAP, FF and FFA residues in aquatic products. Sodium heptane sulfonate was added to the mobile phase, and the target compounds were well separated. Commonly used ion-pairing reagents are sodium heptane sulfonate and sodium lauryl sulfate, and these ion-pairing reagents provide a good separation for the target compounds. Because it is inexpensive, sodium lauryl sulfate is used as an ion pairing reagent in this test. Sodium lauryl sulfate and FFA form weakly polar pairs, which are distributed on the surface of the hydrophobic stationary phase and then eluted by the mobile phase. The buffer system uses phosphate-phosphoric acid and triethylamine to improve peak shape and reduce peak tailing. In this study, the amounts of ion-pairing reagent (sodium lauryl sulfate), buffer system (phosphate-phosphoric acid), and triethylamine were optimized. The effects of 1, 3, 5, and 10 mM sodium lauryl sulfate were investigated. As the concentration increased, the retention time of FFA increased, and a concentration of 3 mM could ensure that FFA eluted first. The effects of 0, 3, 5, 10, and 20 mM NaH_2_PO_4_ are compared in Figure 1a. The retention time of FFA was slightly shorter with increasing NaH_2_PO_4_ concentration, but the use of salt impacted the instrument, and the chromatographic column was easily blocked; thus, considering the FFA retention time and the effect on the instrument, a concentration of 5 mM NaH_2_PO_4_ was selected. The pH impacted the response and retention time of FFA, as shown in Figure 1b. Under neutral conditions, the retention time of FFA was shorter, and decreasing the pH gradually increased the response of FFA (Figure 2) but increased the retention time. When the pH was 5.4, the response of FFA reached its highest value, and further reducing the pH had little effect on the response. However, the retention time was too high, increasing the overall detection time, so a pH of 5.3 ± 0.1 was selected. The amount of triethylamine was also investigated. It was found that 0.01% triethylamine could reduce peak trailing. However, as the amount of triethylamine was increased (0.03%, 0.05%, and 0.1%), the retention time of FFA increased. Based on all these factors, the concentration of triethylamine was set as 0.05%. To separate the targets from impurities, the ratio of the solvents (63:37, 64:36, 65:35, 66:34, *v/v*) in the mobile phase was optimized. When the mobile phase ratio was 64:36 (*v/v*), the targets and impurities were separated, and the peak shapes were good. In summary, the final mobile phase conditions were water (containing 5 mM NaH_2_PO_4_, 3 mM lauryl sodium sulfate, 0.05% triethylamine, adjusted to pH 5.3 ± 0.1) and acetonitrile in a 64:36 (*v/v*) ratio. According to the chemical nature of the ACQUITY UPLC^®^ BEH C18 (2.1 mm × 100 mm, 1.7 μm) column and final mobile phase composition, the elution order and resolution of the target compound were analyzed; TAP was preferentially eluted, followed by FF and then FFA, and examining the fluorescence intensity of the target compound showed that the resolution was greatly improved.

### 2.2. Determination of the Detection Wavelength

Liquid chromatography-fluorescence detection methods are commonly used in veterinary drug residues in animal-derived foods, pesticide residues in agricultural products, and environmental analysis. LC-FLD can detect compounds containing fluorophores, which facilitates the development of simple and rapid methods for the determination of target compounds containing fluorophores. In the literature, when using ultraviolet detectors to identify CAP drugs, the most commonly used detection wavelengths include 220, 223, 224, 225, and 228 nm [15,16,33,34]; when detecting CAP drugs with a fluorescence detector, the excitation and emission wavelengths include 224 and 290 nm, 224 and 295 nm, and 225 and 290 nm, respectively [33,35,36]. However, the optimal detection wavelength for these targets may be different under different detection conditions because of variations in detection devices and experimental conditions. The optimal excitation and emission wavelengths of TAP obtained by fluorescence scanning were 229.8 nm and 285.3 nm, respectively; those of FF were 229.8 nm and 283.9 nm, respectively; and those of FFA were 227.9 nm and 283.9 nm. Therefore, based on the literature and the optimum excitation and emission wavelengths for each target, the optimal detection wavelength for TAP, FF and FFA under the conditions used in this study was simultaneously determined by scanning with a fluorescence detector. An excitation wavelength of 233 nm and an emission wavelength of 284 nm were ultimately selected to simultaneously measure TAP, FF and FFA. These wavelengths ensured both high response values of the targets and no interference from impurities.

### 2.3. Selection and Optimization of the Extraction Solvent and Extraction Method

In the reported literature, the extraction of CAP drugs is typically carried out by liquid-liquid extraction. The most commonly used extractants are acetonitrile, ethyl acetate and a mixture of acetonitrile and ethyl acetate (different proportions of ammonia-containing extractants will provide extracts containing FFA) [33,37,38]. There are few reports on the extraction of CAPs from matrices by automatic extraction equipment-ASE. Only Yang et al. [39] used ASE to extract CAP and FF from aquatic products, and they used static extraction with ethyl acetate at 100 °C for 5 min; their obtained recoveries of the samples were from 90.2% to 109%. Xiao et al. [40] used subcritical water as the extraction solvent to extract traces of CAP, TAP, FF and FFA from poultry tissues using pressurized liquid extractors operating at 150 °C and 100 bar (static extraction, two extraction cycles, 3 min each cycle), and the average recoveries of the four analytes from the samples were 86.8–101.5%. However, a method for simultaneously extracting TAP, FF and FFA from poultry eggs using a modern automatic extraction instrument-ASE instrument has not been reported. In this study, the effects of mixtures of acetonitrile and ammonia (98:2, *v/v*), ethyl acetate and ammonia (98:2, *v/v*) and acetonitrile and ammonia with ethyl acetate (49:49:2, *v/v*/*v*) were compared. The results showed that the above extractants can extract TAP, FF and FFA from the matrix and that the recoveries meet the detection requirements. However, acetonitrile offers better deproteinization, and the substance in eggs that causes the most interference is protein. To improve the detection results, acetonitrile containing ammonia (98:2, *v/v*) was selected as the extractant. Because FFA is a weakly alkaline compound with an amino group, it is more advantageous to extract FFA under alkaline conditions because greater similarity leads to better solubility. Under optimized ASE conditions, this study compared the effects of different ratios of extractants (acetonitrile: ammonia = 99:1, 98:2, 97:3, 96:4, and 95:5, *v/v*) on poultry egg recovery. Table 1 shows that as the proportion of ammonia increased, the recoveries of FFA also gradually increased; however, when the content of ammonia exceeded 2% of the total volume, the recoveries of FFA decreased, and excessive ammonia also reduced the recoveries of TAP and FF. In summary, the extraction outcome with acetonitrile:ammonia (98:2, *v/v*) was best, and the recoveries of all targets were above 91.0%.

In this study, under the conditions of acetonitrile:ammonia (98:2, *v/v*) as an extractant, the effects of ultrasonic extraction, vortex oscillation extraction, vortex oscillation + ultrasonic extraction and ASE extraction were compared. Table 2 shows that compared to those with other sample preparation processes, the recoveries with ASE extraction were best; in addition, the ASE method saves time and is suitable for batch processing of samples. The time required to prepare a sample with ASE (15 min) was half that required for vortexing + ultrasonic extraction (30 min). Moreover, using ASE to process samples avoided contact between the experimenter and the reagent, which is more consistent with the detection requirements of health and environmental protection. Therefore, this study ultimately selected ASE for sample extraction.

### 2.4. Optimization of the ASE Method

**Figure 3:**
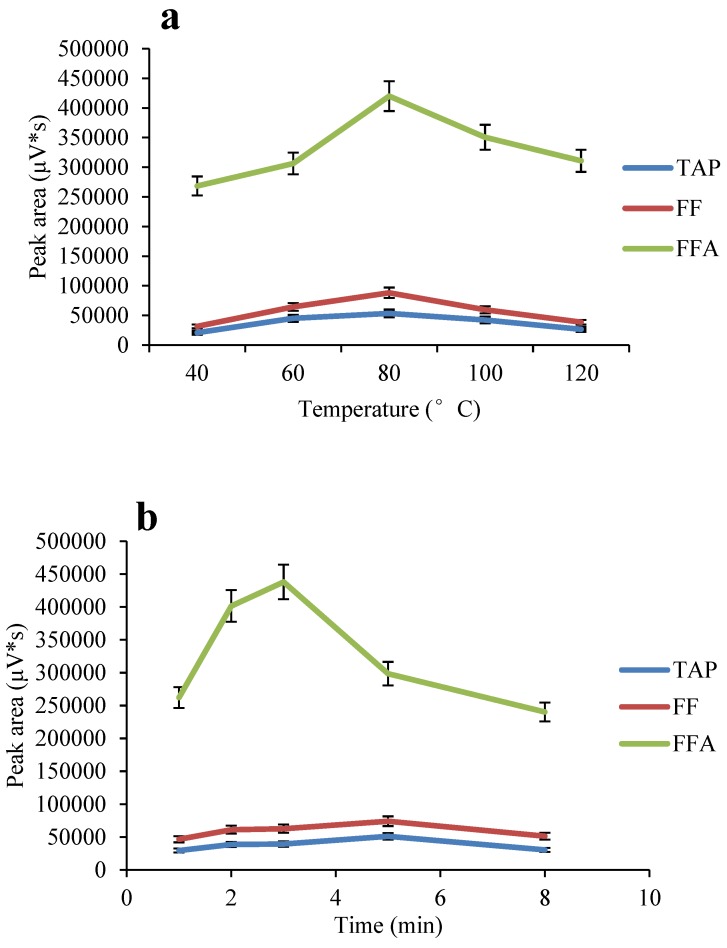
Effects of temperature (**a**) and time (**b**) on ASE extraction.

The sample extraction step is often considered to be the bottleneck of the entire analytical process. To simplify the pretreatment of the sample and improve the efficiency of sample preparation, a variety of pretreatment methods have been developed. Since the introduction of ASE in 1995, it has rapidly become an acceptable alternative to traditional extraction methods. ASE uses high-temperature and high-pressure conditions, which result in greatly improved extraction efficiency. This study explored the effects of various operating parameters (temperature, time, volume of solvent used and so on) on ASE performance. The effects of different temperatures (40, 60, 80, 100 and 120 °C) on the recoveries of the targets were studied. The recoveries of the targets gradually increased with increasing extraction temperature. When the temperature exceeded 80 °C, the recoveries decreased (Figure 3a). Studies have shown that increasing the time of static extraction provides the target sufficient time to diffuse into the extraction solvent, improving the efficiency of the extraction. However, this study compared different static extraction times (2, 3, 5, and 8 min) and found that for TAP and FF, static extraction for 5 min provides high recoveries (>90%), while the recovery of FFA decreases with increasing static extraction time (Figure 3b). As shown in Figure 3b, 3 min and 5 min had little effect on TAP and FF extraction, and a high recovery rate for FFA was observed when the static extraction time was 3 min. Therefore, to ensure that the recoveries of all the targets met the requirements of detection under these ASE conditions, 3 min was selected as the static extraction time. This study also explored the effect of the number of static extraction cycles and the volume of the extractant on the extraction outcome. Extraction of the target twice provided a better recovery than one extraction cycle. When the volume of the extractant was 40% by volume, the target could be efficiently extracted, so there was no need to increase the amount of extractant. In summary, the final ASE conditions were 80 °C, 1500 psi, 40% pool volume, static extraction for 3 min, and two static extraction cycles.

### 2.5. Bioanalytical Method Validation

In the blank poultry eggs, TAP was spiked at a concentration from limit of quantification (LOQ)-250 μg/kg, and FF and FFA were added at LOQ-400 μg/kg. The peak area was correlated with the spiked concentration of the analyte, and the linearity was good. The linear equations, linear ranges and coefficients of determination of TAP, FF and FFA in poultry eggs are shown in Table 3.

The recoveries and precisions of TAP, FF and FFA in different blank poultry egg samples are shown in Table 4 and Table 5, respectively. As shown in Table 4 and Table 5, the recoveries of TAP, FF and FFA in poultry eggs were 80.1%–98.6%, the relative standard deviations (RSDs) were 1.2%–4.3%, the intraday RSDs were 1.2%–5.5%, and the interday RSDs were 1.8%–6.6%. According to the EU 2002/675/EC resolution and the FDA [41,42], the acceptable range of recoveries for multidrug residue testing procedures is 70–120%. The average recoveries of TAP, FF and FFA from different blank poultry egg samples were all above 80.0%, which are consistent with the EU’s requirements for the recoveries of analytes. The limits of detection (LODs) and LOQs of TAP, FF and FFA in different blank poultry egg samples using the optimized pretreatment method and instrument analysis method are shown in Table 3. The LODs of TAP, FF and FFA were 3.3–3.4 μg/kg, 4.7–4.9 μg/kg and 1.8–1.9 μg/kg, respectively, and the LOQs were 9.7–9.9 μg/kg, 10.5–11.7 μg/kg and 4.3–4.8 μg/kg, respectively.

**Figure 4:**
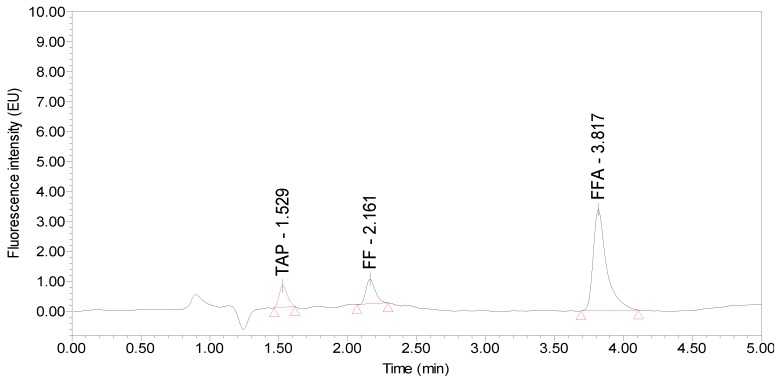
Chromatogram of the standards (25 µg/kg TAP and 50 µg/kg FF and FFA standards).

**Figure 5:**
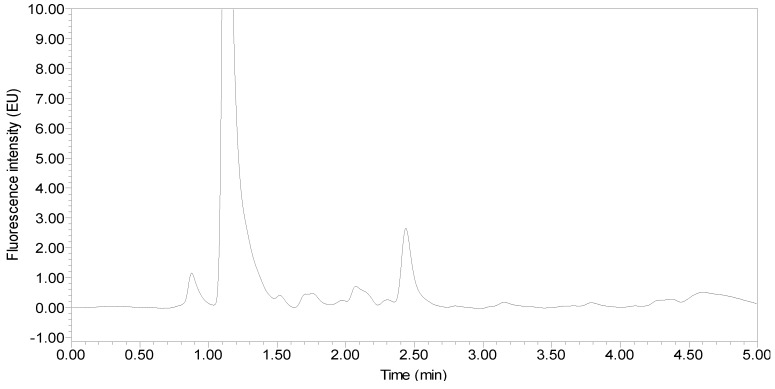
Chromatogram of the blank hen egg.

**Figure 6:**
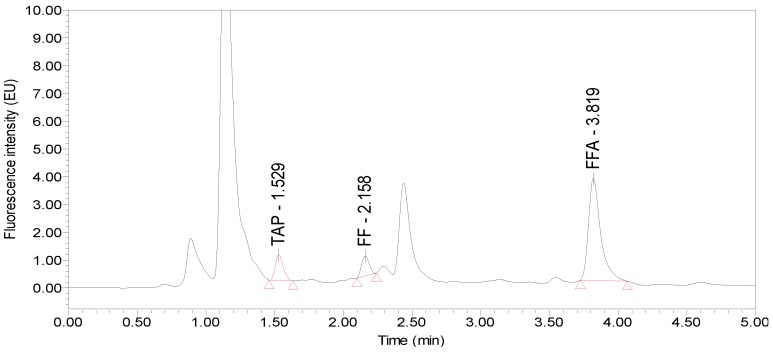
Chromatogram of the blank hen egg spiked with 25 µg/kg TAP and 50 µg/kg FF and FFA standards.

Under the optimized UPLC-FLD conditions, the retention times of TAP, FF and FFA from different poultry eggs were 1.50, 2.10 and 3.80 min, respectively. The peak shapes were good, and the blank samples had no interference peaks around these retention times. Taking hen egg samples as an example, the chromatograms of the standards, blank hen egg samples and blank hen egg samples spiked with standards are shown in Figure 4, Figure 5 and Figure 6.

### 2.6. Real Sample Analysis

Our newly developed detection method was applied to the evaluation of real samples. A total of 150 commercial poultry egg samples (30 hen eggs, 30 duck eggs, 30 goose eggs, 30 pigeon eggs and 30 quail eggs) from a local supermarket were analyzed by the described method. Only hen eggs (34 and 20 μg/kg) and duck eggs (44 and 18 μg/kg) were found to contain FF and FFA residues, and none of the samples exceeded the MRL of 100 μg/kg (EU standard). From these data, we can evaluate the applicability and reliability of the newly developed method. Thus, this new UPLC-FLD method can be applied to the quantification of these drugs in poultry egg samples.

## 3. Materials and Methods

### 3.1. Chemicals and Reagents

TAP (99.0% purity), FF (99.0% purity) and FFA (99.8% purity) standards were obtained from Dr. Ehrenstorfer GmbH (Augsburg, Germany). HPLC-grade acetonitrile and triethylamine were purchased from EMD Millipore Company Inc. (Billerica, MA, USA) and Tedia Company Inc. (Fairfield, OH, USA), respectively. Other reagents were of analytical grade and were supplied by Sinopharm Chemical Reagent Co. Ltd. (Shanghai, China).

### 3.2. Standard and Working Solutions

Stock solutions of TAP and FF at concentrations of 400.0 mg/L and of FFA at a concentration of 100.0 mg/L were prepared by dissolving TAP, FF, and FFA (initially dissolved in 1 mL of ultrapure water), respectively, in acetonitrile. Working standard solutions of TAP, FF, and FFA at different concentrations were prepared by diluting the stock solutions with acetonitrile-water (36:64, *v/v*). The stock solutions were stable for five months at −70 °C. Fresh working solutions were prepared by dilution of the stock solution before use.

### 3.3. UPLC-FLD Instrumentation and Conditions

A Waters ACQUITY UPLC System and a Waters fluorescence detector (Waters Corp., Milford, MA, USA) were used. The separation was achieved on an ACQUITY UPLC^®^ BEH C_18_ (2.1 mm × 100 mm, 1.7 μm) chromatographic column. The column temperature was maintained at 30 °C. The injection volume was 10 μL. The analysis was carried out using acetonitrile and 0.005 mol/L NaH_2_PO_4_ solution containing 0.003 mol/L sodium dodecyl sulfate and 0.05% triethylamine with pH (FE20, METTLER TOLEDO, Shanghai, China) adjusted to 5.3 ± 0.1 with 85% phosphoric acid as the mobile phase (36:64, *v/v*) at a flow rate of 0.2 mL/min, and the excitation and emission wavelengths were 233 nm and 284 nm, respectively.

### 3.4. Sample Preparation

#### 3.4.1. ASE Extraction

Homogeneous blank poultry egg samples (2.0 ± 0.02 g) were accurately weighed in a mortar and then ground with the appropriate amount of diatomite. The samples were then transferred into a 22 mL extraction tank, placed on the mechanical arm of the accelerated solvent extractor for extraction, and the extractant was acetonitrile:ammonia (98:2, *v/v*). The extraction method and parameters were as follows: extraction pressure of 1500 psi, extraction temperature of 80 °C, extraction time of 3 min, solvent flush of approximately 40% of the tank volume, and two cycles of static extraction. Finally, the extract was collected and left to stand.

#### 3.4.2. Ultrasonic Extraction

Homogeneous blank poultry egg samples (2.0 ± 0.02 g) were accurately weighed into 50 mL polypropylene centrifuge tubes, and 1 mL of 30% acetonitrile solution was added. The solution was vortexed, and then 10 mL of acetonitrile:ammonia (98:2, *v/v*) was added. After ultrasonic extraction for 15 min via an ultrasonic cleaning machine (P300H Elma, Konstanz, Germany), the solution was centrifuged at 8000× *g* for 10 min in a desktop high-speed refrigerated centrifuge (5810R, Eppendorf, Hamburg, Germany), and the supernatant was transferred to a 50 mL propylene centrifuge tube. The extraction process was repeated a second time, and the two extracts were combined for further analysis.

#### 3.4.3. Vortex Shock Extraction

Homogeneous blank poultry egg samples (2.0 ± 0.02 g) were accurately weighed into 50 mL polypropylene centrifuge tubes, and 1 mL of 30% acetonitrile solution was added. The solution was vortexed on a vortex oscillator (G560E, Scientific Industries Ltd., Bohemia, New York, NY, USA), 10 mL of acetonitrile:ammonia (98:2, *v/v*) was added, and the sample was vortexed for 2 min. The solution was centrifuged at 8000× *g* for 10 min and then transferred to 50 mL polypropylene centrifuge tubes. The extraction process was repeated a second time, and the two extracts were combined for further analysis.

#### 3.4.4. Vortex Oscillating + Ultrasonic Extraction

Homogeneous blank poultry egg samples (2.0 ± 0.02 g) were accurately weighed into 50 mL polypropylene centrifuge tubes, and 1 mL of 30% acetonitrile solution was added. The samples were vortex mixed, 10 mL of acetonitrile:ammonia (98:2, *v/v*) was added, and they were vortex mixed for an additional 2 min. Then, they were subjected to ultrasonication for 15 min. After centrifugation at 8000× *g* for 10 min, the supernatant was transferred to a 50 mL polypropylene centrifuge tube. The extraction process was repeated a second time, and the two extracts were combined for further analysis.

#### 3.4.5. Sample Purification

The collected extract was concentrated to near dryness in a centrifugal concentrator, and the residue was dissolved in 1 mL of acetonitrile and then defatted with acetonitrile-saturated n-hexane (hen, duck, and goose whole egg and yolk with 10 × 10 mL; egg white with 5 mL; and pigeon eggs and quail eggs with 8 × 8 mL). After vortexing for 1 min, the upper layer was left to rest for 5 min. The centrifuge tube was placed into the centrifugal concentrator to concentrate the lower liquid layer to dryness. The dried sample was reconstituted with 2.0 mL of the mobile phase, vortexed for 1 min, and centrifuged at 12,000× *g* for 15 min. The supernatant was passed through a 0.22 μm PVDF needle, and the filtrate was analyzed by UPLC-FLD.

### 3.5. Method Validation

This optimized method was validated according to the requirements defined by the EU and the FDA [41,42]. The selectivity of the method was estimated by preparing and analyzing 20 blank and spiked samples. The probable interferences from endogenous substances were assessed based on the chromatograms of blank and spiked poultry egg samples. The sensitivity of the method was assessed in terms of the LODs and LOQs. The LODs were defined by the concentration of each of the three analytes in the sample matrix that resulted in a signal-to-noise (S/N) ratio of 3:1. The LOQs were defined as the lowest concentration on the calibration curve of each of the three analytes giving a S/N ratio of 10:1.

The calibration curves were prepared based on the peak areas and the concentrations of the working solutions. A series of working standard solutions at concentrations of LOQ, 20.0, 50.0, 100.0, 150.0, 200.0, and 250.0 µg/kg for TAP; LOQ, 20.0, 50.0, 100.0, 200.0, 300.0, and 400.0 µg/kg for FF; and LOQ, 10.0, 20.0, 50.0, 100.0, 200.0, and 400.0 µg/kg for FFA were prepared by diluting the stock solutions with extract of blank sample matrix, and then these solutions were analyzed by the UPLC-FLD method.

The accuracy and precision of the method were evaluated by determining the recoveries of TAP, FF and FFA in poultry egg samples at concentrations of LOQ, 0.5 MRL, 1.0 MRL and 2.0 MRL. The recovery of the method was calculated by comparing the determined concentrations of samples to their theoretical concentrations.

## 4. Conclusions

In this study, an ASE method for the extraction of TAP, FF and FFA from poultry eggs was established. This extraction method has high extraction efficiencies and high recoveries (>80.0%), and the entire process is automated. This UPLC-FLD method for the quantitative determination of TAP, FF and FFA is accurate and sensitive. The parameters of this method were shown to meet the requirements of the Chinese Ministry of Agriculture, the EU and the FDA for the detection of veterinary drug residues.

## Figures and Tables

**Table 1:** Effects of different ratios of extraction reagents (acetonitrile:ammonia) for ASE on the recoveries of 25 µg/kg TAP and 50 µg/kg FF and FFA from poultry eggs (%) (*n* = 6).

Matrix	Analyte	Extraction Reagents (acetonitrile:ammonia, *v/v*)				
		99:1	98:2	97:3	96:4	95:5
Hen eggs	TAP	93.4 *±* 1.8	92.8 *±* 1.1	89.4 *±* 2.3	81.6 *±* 2.2	73.9 *±* 2.0
	FF	92.6 *±* 2.7	93.2 *±* 3.0	84.2 *±* 2.2	74.3 *±* 1.9	69.6 *±* 2.0
	FFA	86.9 *±* 2.1	92.4 *±* 2.2	88.4 *±* 3.1	78.8 *±* 2.9	69.3 *±* 1.9
Duck eggs	TAP	90.2 *±* 2.3	91.3 *±* 2.3	85.3 *±* 2.4	80.8 *±* 2.4	70.3 *±* 2.2
	FF	93.0 *±* 1.8	91.2 *±* 2.5	84.2 *±* 2.0	74.6 *±* 1.9	68.7 *±* 2.3
	FFA	85.0 *±* 2.5	93.2 *±* 1.8	84.3 *±* 2.3	73.6 *±* 1.9	61.5 *±* 1.9
Goose eggs	TAP	90.7 *±* 2.2	93.4 *±* 2.6	86.7 *±* 2.4	75.8 *±* 2.7	67.9 *±* 2.0
	FF	93.1 *±* 1.8	91.3 *±* 1.9	87.3 *±* 2.3	71.4 *±* 2.2	60.4 *±* 2.4
	FFA	85.0 *±* 1.8	92.3 *±* 2.0	84.1 *±* 1.8	73.0 *±* 1.9	63.8 *±* 2.2
Pigeon eggs	TAP	92.4 *±* 2.6	92.6 *±* 2.1	82.3 *±* 3.1	75.5 *±* 2.4	63.2 *±* 2.3
	FF	92.3 *±* 2.4	93.1 *±* 2.0	83.8 *±* 1.9	76.1 *±* 3.0	61.0 *±* 2.2
	FFA	88.2 *±* 2.2	96.3 *±* 2.1	86.5 *±* 2.7	73.1 *±* 2.1	64.8 *±* 2.3
Quail eggs	TAP	91.3 *±* 2.3	92.8 *±* 2.3	87.3 *±* 2.0	70.8 *±* 1.9	65.7 *±* 2.1
	FF	92.7 *±* 2.2	94.2 *±* 2.5	86.2 *±* 2.5	74.5 *±* 2.4	68.5 *±* 2.5
	FFA	86.5 *±* 1.8	93.0 *±* 1.9	84.7 *±* 2.5	76.2 *±* 1.9	70.4 *±* 1.8

**Table 2:** Effects of different extraction methods on the recoveries of 25 µg/kg TAP and 50 µg/kg FF and FFA from poultry eggs (%) (*n* = 6).

Matrix	Analyte	Extraction Method			
		Ultrasonic	Vortex Oscillation	Vortex Oscillation + Ultrasonic	ASE
Hen egg	TAP	34.4 *±* 2.5	78.6 *±* 2.6	88.3 *±* 2.2	92.8 *±* 2.4
	FF	37.1 *±* 2.3	81.5 *±* 3.1	92.0 *±* 2.7	96.0 *±* 2.3
	FFA	54.4 *±* 2.2	84.0 *±* 2.9	91.9 *±* 2.8	93.3 *±* 2.0
Duck eggs	TAP	30.9 *±* 2.0	70.0 *±* 2.7	84.9 *±* 2.2	90.2 *±* 1.9
	FF	33.4 *±* 2.5	79.5 *±* 2.7	89.7 *±* 2.4	92.5 *±* 2.1
	FFA	49.0 *±* 2.4	74.4 *±* 3.2	86.0 *±* 2.9	91.7 *±* 2.2
Goose eggs	TAP	31.3 *±* 2.9	71.9 *±* 3.0	82.7 *±* 2.6	93.3 *±* 1.9
	FF	35.6 *±* 2.7	72.0 *±* 2.6	88.4 *±* 3.0	92.4 *±* 2.5
	FFA	40.5 *±* 3.4	77.0 *±* 2.8	85.0 *±* 2.4	90.4 *±* 2.1
Pigeon eggs	TAP	34.4 *±* 3.0	69.9 *±* 2.7	85.3 *±* 2.9	86.9 *±* 2.2
	FF	34.7 *±* 2.6	70.0 *±* 3.2	87.0 *±* 2.6	89.0 *±* 2.4
	FFA	40.7 *±* 2.8	72.6 *±* 2.9	80.3 *±* 2.3	95.1 *±* 1.6
Quail eggs	TAP	36.1 *±* 2.9	72.1 *±* 2.8	83.5 *±* 2.7	90.1 *±* 2.0
	FF	30.1 *±* 3.0	73.4 *±* 2.3	88.5 *±* 3.3	92.6 *±* 2.4
	FFA	45.5 *±* 2.8	69.7 *±* 3.1	83.7 *±* 2.5	92.7 *±* 2.7

**Table 3:** The linear ranges, linear regression equations, determination coefficients, LODs and LOQs of TAP, FF and FFA from poultry eggs.

Matrix	Analyte	Linear Range (µg/kg)	Linear Regression Equation	Determination Coefficient (R^2^)	LOD (µg/kg)	LOQ (µg/kg)
Hen eggs	TAP	9.7–250.0	y = 1030.2x + 295.52	0.9996	3.3	9.7
	FF	10.5–400.0	y = 737.43x + 714.06	0.9998	4.7	10.5
	FFA	4.3–400.0	y = 3020.3x + 164.01	0.9998	1.8	4.3
Duck eggs	TAP	9.9–250.0	y = 562.84x + 634.05	0.9997	3.4	9.9
	FF	11.7–400.0	y = 844.1x + 616.34	0.9997	4.9	11.7
	FFA	4.7–400.0	y = 4495.8x + 520.7	0.9996	1.9	4.7
Goose eggs	TAP	9.8–250.0	y = 618.73x + 139.95	0.9996	3.4	9.8
	FF	11.2–400.0	y = 713.68x + 738.78	0.9996	4.8	11.2
	FFA	4.7–400.0	y = 3081.1x – 258.32	0.9997	1.9	4.7
Pigeon eggs	TAP	9.9–250.0	y = 683.44x + 543.48	0.9998	3.4	9.9
	FF	11.2–400.0	y = 762.9x + 761.11	0.9993	4.8	11.2
	FFA	4.8–400.0	y = 4019.1x + 490.45	0.9998	1.9	4.8
Quail eggs	TAP	9.7–250.0	y = 667.44x + 483.91	0.9993	3.3	9.7
	FF	10.6–400.0	y = 753.98x – 193.97	0.9999	4.7	10.6
	FFA	4.6–400.0	y = 4824.6x – 229.06	0.9994	1.8	4.6

**Table 4:** Recoveries and precisions of TAP, FF and FFA from spiked blank hen eggs, duck eggs and goose eggs.

Matrix	Analyte	Spiking Level (µg/kg)	Recovery (%) (*n* = 6)	RSD (%) (*n* = 6)	Intraday RSD (%) (*n* = 6)	Interday RSD (%) (*n* = 18)
Hen eggs	TAP	9.7	85.6 ± 1.8	2.1	2.6	3.6
		25	90.5 ± 2.7	3.0	3.7	5.0
		50 ^α^	92.7 ± 1.5	1.6	2.5	3.1
		100	91.5 ± 2.0	2.2	2.5	2.9
	FF	10.5	84.9 ± 3.4	4.0	4.7	5.1
		50	90.2 ± 2.5	2.8	3.8	4.8
		100 ^α^	93.5 ± 2.6	2.8	4.0	4.3
		200	94.9 ± 2.7	2.8	2.3	3.4
	FFA	4.3	86.7 ± 3.6	4.2	3.9	5.3
		50	91.5 ± 1.8	2.0	3.1	3.6
		100 ^α^	96.7 ± 3.5	3.6	3.2	2.8
		200	98.0 ± 1.8	1.8	2.4	2.7
Duck eggs	TAP	9.9	84.8 ± 2.0	2.4	3.9	4.2
		25	93.8 ± 1.8	1.9	3.6	4.6
		50 ^α^	94.5 ± 2.4	2.5	1.9	2.6
		100	92.8 ± 1.1	1.2	2.4	2.4
	FF	11.7	85.1 ± 1.6	1.9	1.2	2.4
		50	89.4 ± 2.0	2.2	1.8	1.8
		100 ^α^	94.8 ± 1.5	1.6	2.5	3.6
		200	94.5 ± 3.3	3.5	2.6	4.1
	FFA	4.7	87.5 ± 1.9	2.2	3.1	3.7
		50	96.5 ± 2.2	2.3	3.1	3.4
		100 ^α^	96.9 ± 1.7	1.8	2.7	3.6
		200	96.1 ± 1.7	1.8	3.1	3.3
Goose eggs	TAP	9.8	85.5 ± 1.7	2.0	2.5	2.9
		25	93.3 ± 2.1	2.3	3.5	4.0
		50 ^α^	93.1 ± 2.9	3.1	4.3	5.4
		100	94.5 ± 4.0	4.2	3.9	4.0
	FF	11.2	80.7 ± 3.5	4.3	5.2	6.2
		50	93.9 ± 2.2	2.3	2.7	3.1
		100 ^α^	95.2 ± 1.5	1.6	2.2	3.0
		200	95.2 ± 2.5	2.6	3.1	3.4
	FFA	4.7	83.9 ± 2.8	3.3	3.5	4.1
		50	93.7 ± 2.5	2.7	3.2	3.8
		100 ^α^	94.5 ± 2.0	2.1	3.2	3.5
		200	96.1 ± 2.6	2.7	3.3	4.4

Note: α. Maximum Residue Limits.

**Table 5:** Recoveries and precisions of TAP, FF and FFA from spiked blank pigeon eggs and quail eggs.

Matrix	Analyte	Spiking Level (µg/kg)	Recovery (%) (*n* = 6)	RSD (%) (*n* = 6)	Intraday RSD (%) (*n* = 6)	Interday RSD (%) (*n* = 18)
Pigeon eggs	TAP	9.9	80.1 ± 2.5	3.1	2.5	3.6
		25	93.6 ± 3.3	3.5	4.7	5.4
		50 ^α^	92.3 ± 2.4	2.6	2.7	3.2
		100	94.4 ± 3.3	3.5	2.2	4.3
	FF	11.2	84.4 ± 2.2	2.6	3.2	3.3
		50	95.9 ± 2.5	2.6	4.4	4.2
		100 ^α^	97.9 ± 4.1	4.2	3.3	5.0
		200	98.6 ± 2.8	2.8	3.1	3.1
	FFA	4.8	85.7 ± 2.0	2.3	3.8	4.1
		50	94.0 ± 2.3	2.4	2.9	3.2
		100 ^α^	95.7 ± 3.2	3.3	4.2	5.4
		200	97.7 ± 1.8	1.8	2.3	3.7
Quail eggs	TAP	9.7	84.1 ± 3.0	3.6	3.9	4.0
		25	95.4 ± 2.0	2.1	3.3	3.9
		50 ^α^	93.4 ± 2.8	3.0	3.9	4.4
		100	96.3 ± 2.2	2.3	3.7	5.4
	FF	10.6	86.5 ± 3.5	4.0	5.5	5.5
		50	94.9 ± 2.1	2.2	2.7	3.8
		100 ^α^	95.9 ± 3.1	3.2	4.0	4.6
		200	96.7 ± 2.3	2.4	3.2	3.4
	FFA	4.6	87.5 ± 2.7	3.1	3.7	4.1
		50	96.0 ± 3.0	3.1	4.7	5.6
		100 ^α^	95.5 ± 3.4	3.6	3.5	5.5
		200	96.2 ± 3.6	3.7	5.4	6.6

Note: α. Maximum Residue Limits.
